# The Power of a Positive Human–Animal Relationship for Animal Welfare

**DOI:** 10.3389/fvets.2020.590867

**Published:** 2020-11-09

**Authors:** Jean-Loup Rault, Susanne Waiblinger, Xavier Boivin, Paul Hemsworth

**Affiliations:** ^1^Institute of Animal Welfare Science, University of Veterinary Medicine, Vienna, Austria; ^2^Unité Mixte de Recherche sur les Herbivores, Université Clermont Auvergne, INRA, VetAgro Sup, UMR Herbivores, Saint-Genès-Champanelle, France; ^3^Animal Welfare Science Centre, Faculty of Veterinary and Agricultural Sciences, The University of Melbourne, Parkville, VIC, Australia

**Keywords:** agency, domestic, interaction, inter-species, perception, positive welfare, welfare assessment, well-being

## Abstract

Domestic animals often seek and enjoy interacting with humans. Positive human–animal relationships can elicit positive emotions and other positive welfare outcomes. Nevertheless, our understanding of the underlying processes that govern the positive perception of humans by animals is incomplete. We cover the potential mechanisms involved in the development and maintenance of positive human–animal relationships from the perspective of the animal. This encompasses habituation, associative learning, and possibly attachment or bonding based on communication and social cognition. We review the indicators from the literature to assess a positive human–animal relationship. We operationally define this positive relationship as the animal showing voluntary approach and spatial proximity (seeking) and signs of anticipation, pleasure, relaxation, or other indicators of a rewarding experience from interacting with the human. For research, we recommend accounting for the baseline human–animal relationship in the animal's everyday life, and incorporating a control treatment rather than only comparing positive to negative interaction treatments. Furthermore, animal characteristics, such as previous experience, genetics, and individual predisposition, as well as contextual characteristics related to the social and physical environment, may modulate the perception of humans by animals. The human–animal relationship is also influenced by human characteristics, such as the person's familiarity to the animal, attitudes, skills, and knowledge. We highlight implications for current practices and suggest simple solutions, such as paying attention to the animal's behavioral response to humans and providing choice and control to the animal in terms of when and how to interact with humans. Practical applications to achieve a positive perception of humans could be better utilized, such as by incorporating training principles, while keeping in mind trust and safety of both partners. Overall, there is growing evidence in the scientific literature that a positive human–animal relationship can bring intrinsic rewards to the animals and thereby benefit animal welfare. Further research is needed on the underlying processes to establish an effective positive human–animal relationship, especially in regard to the type, frequency, and length of human interaction necessary. In particular, the importance of providing animals with a sense of agency over their interactions with humans remains poorly understood.

## Introduction

The human–animal relationship (HAR) is an important determinant of animal welfare (1–3). Numerous studies have demonstrated the detrimental effects of a negative HAR on animal and human welfare, that is, productivity, companionship, health (4). A negative HAR can impair animal welfare with negative consequences on the animal's productivity, health, and welfare, primarily through fear as an underlying mechanism (1, 5). In comparison, the benefits of a positive HAR for animal welfare are poorly understood and appreciated. Domestic animals often seek and enjoy interacting with humans, beyond depending on humans for food (6–9). Animals may perceive interacting with humans *per se* as rewarding (5, 10, 11).

This review compiles the recent knowledge of the welfare benefits for animals of interacting positively with humans and provides recommendations to assess and utilize a positive HAR. We focus on the HAR from the perspective of the non-human animal (hereby referred to as “animal”) unless stated otherwise. For the HAR from the human's perspective, we refer the readers to other reviews (5, 12, 13). We restrict the scope of this article to domesticated species, primarily farm and companion animals, because they have been (and still are) selected over thousands of years with a major influence on their response to humans (9, 14), and most domestic animals experience frequent interactions with humans. Notwithstanding, animals from other species are also able to develop positive relationships with humans, for instance, animals kept in zoos (1, 15, 16) or laboratories (17, 18), and therefore examples on these species are included where relevant.

## Mechanisms for the Formation of a Positive HAR

### Definitions

A positive HAR can be defined conceptually based on a positive perception by the animal of the human. Because perception is challenging to assess practically, a positive HAR can be defined operationally in that the animal shows voluntary approach and spatial proximity (seeking) and signs of anticipation, pleasure, relaxation, or other indicators of a rewarding experience arising from interacting with the human. Fear of humans prevents a positive perception of humans, but low or no fear is in itself not a sufficient condition. A positive HAR brings beneficial short-term [e.g., positive emotions (19)] and long-term [e.g., stress resilience (20)] welfare outcomes for the animal when or after interacting with the human (see section Implications for Practice).

### Habituation

HARs are most often referred to in the context of fear of humans (21), although positive HARs have received increased attention recently (1–3, 22, 23). This questions whether a positive HAR can be understood, as for negative HAR, solely as a consequence of a reduction in the fear response to humans or an absence of fear. When a stimulus is unfamiliar, fear is usually the default response. Fear of humans can be reduced through habituation, defined as a reduction in response resulting from repeated exposure to a stimulus (24). Although it can reduce fear of humans by leading to a neutral response, habituation is insufficient to reach a positive HAR. This non-associative learning process can occur by direct exposure, but also be facilitated or inhibited by social learning or transmission from the dam or other animals (25, 26).

### Interactions vs. Relationship

The formation of a relationship is a progressive process, reinforced upon subsequent interactions. This highlights the difference between an interaction and a relationship based on a single vs. multiple events between two individuals, respectively (27), with a relationship developing on the basis that animals are able to memorize and predict future interactions with humans (28, 29). We focus on the relationship rather than interactions because the HAR is more relevant for welfare because of its long-lasting and integrative nature (i.e., comprising past interactions, present, and predicting future ones). Of course, there is a link between interactions and the resulting relationship. In particular, the formation of a positive HAR may be jeopardized by negative interactions, even when the occurrence of positive interactions far outweighs negative interactions (30). However, a strong or high-quality HAR may endure deviation from positive interactions or be more resilient to aversive events (31, 32). The time at which a relationship is formed remains difficult to determine, but it can be defined as the time at which the animal forms expectations of its interaction with humans.

### Associative Learning

Associative learning can accelerate the formation of a relationship, by the animal associating humans with positive aspects either through classical conditioning (the human presence itself or its concurrent association with a positive event) or operant conditioning (interacting with the human leads to positive consequences). A positive HAR can be established by human contact that is inherently rewarding such as through stroking or brushing [dog (33), sheep (6, 7), cattle (11, 34, 35), pig (36, 37)] or play interactions [dog (38), cat (39)]. However, not all individuals react in the same manner to putative positive interactions. For example, previous interactions affect the way animals perceived human contact [pig (32, 40)], supporting a role for ontogeny. Furthermore, animals from different genetic origins can also perceive stroking by humans differently [dog (9), sheep (41)], supporting a role for phylogeny. The role of potential modulating factors such as individual differences (e.g., personality) and affective states should be investigated further. It should be noted that a positive HAR cannot simply be explained by food or other resources provided by humans, although food can facilitate the development of a positive HAR [sheep (7), cow (42), pig (8), cat (39)].

### Bonding

In addition to associative learning processes, a number of phenomena have been proposed to explain the formation of a positive HAR, in particular aspects relevant to social bonding and related constructs. Familiarity with a human does not necessarily equate to a positive HAR, although it may be conducive to it given that repeated non-aversive exposure can facilitate positive appraisal [“the mere exposure” effect (43)]. The attachment theory has been used in the context of the HAR (26), originating from the study of infant–parent relationships and defined as an affectional bond binding the individuals together in space and enduring over time (44). Without any obvious reinforcement, and because animals need to feel safe and have a basis from which they can explore their world, attachment can occur with familiar individuals such as the mother, peers, other conspecifics, and even individuals from other species such as humans (26). These animals calm quickly after a short period of social isolation when in the presence of a familiar human [dog (33, 45), cat (46), hand-reared lamb (26, 47), pig (48)]. The socialization process (49, 50) may also play a role in the context of the HAR, through learning how to behave toward others. Indeed, a successful relationship encompasses both the intent by the animal and the human to interact, as well as competent social skills relying on sociocognitive and communicative abilities (see other contributions in this Special Issue).

## Assessment of a Positive Human-Animal Relationship

The HAR can be observed either through observations of spontaneous interactions (i.e., without interference) or through stimulus-evoked situations and tests that investigate the HAR in a more systematic way.

### Indicators

A number of biological changes can occur before, during, and/or after interactions with humans (Table 1). Most of these indicators are based on features of the interactions, indirectly reflecting the HAR. Some indicators can distinguish different qualities of the relationship (e.g., evaluate which animals have a better relationship than others), but it is generally difficult to set a threshold where a positive HAR starts, apart from some indicators that clearly reflect a positive HAR. The assessment of a positive HAR requires a holistic analysis, given that several indicators need to be considered together for a full understanding. Care is required in assessing a positive HAR because, for example, the motivation to interact with humans may at the time be conflicting with other motivations, and some indicators of a positive HAR are species-specific.

**Table 1:** List of indicators of a positive human–animal relationship.

**Category**	**Indicator**	**Examples of measures**	**Direction of change**	**Specificity^1^**	**Key references**
Movement/location	Approach^2^	Latency to approach/touch human	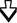	Conditional	(22)
		Direction of movement relative to human	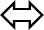	Conditional	(22)
	Spatial proximity	Time in proximity of human	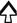	Conditional	(30, 51)
Expressive behaviors	Vocalizations^2^	Yapping, purring, chirping, grunting, other low-frequency vocalizations	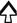	Conditional	(46, 48)
	Ear posture^2^	Relaxed ears	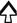	Yes	(52–58)
	Tail posture	Tail low, or wagging	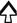	Conditional	(59)
	Body posture	Relaxed, laying down near the human	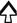	Yes	(48)
	Facial expression	Muscle movement, change in facial display, closed/half-closed eyes	?	?	(60, 61)
Qualitative behavior assessment		Positively valenced factors	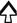	Yes	(62)
Preference for human^2^		Choice or motivation for specific human(s) over other stimuli, memory of humans	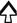	Conditional	(28, 32, 36, 48, 63, 64)
Characteristics of the interaction	Initiating physical interaction^2^	Exposing a body area	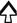	Conditional	(35, 37, 52, 65)
		Solicitation behaviors like nudging, scratching, play bow	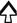	Yes	(66)
	Interaction features	Frequency or duration of physical contact, eye gaze	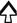	Conditional	(67)
	Reaction to human contact	Acceptance of stroking or touch	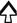	Conditional	(68)
	Behavioral synchrony	Temporal synchrony of behavioral exchanges/movement	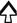	?	(69)
	Behavioral matching	Complementarity, reciprocity of behavioral exchanges	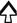	?	(70)
During or postinteraction effects	Relaxation^2^	Duration or shorter latency to rest or sleep, rumination	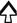	Yes	(55, 71–74)
	Exploration^2^	Secure base exploration	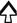	Conditional	(26, 50)
Postinteraction behavioral changes	Separation distress^2^	Searching behavior, distress vocalizations, or contact calls	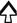	Conditional	(47, 51, 75, 76)
Physiological indicators	Oxytocin^2^	Hormone concentration or change	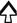	Conditional	(77)
	Heart rate^2^	Heart rate beat per min	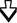	Conditional	(54, 78, 79)
	Parasympathetic activity^2^	Heart rate variability: high-frequency band, RMSSD	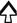	Conditional	(54, 78, 79)
Cognitive and neurobiological measures	Cognitive bias	Positive judgment of ambiguous cues	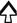	Conditional	(40, 80)
	Neurobiology	EEG, fNRIS, neuroimaging (e.g., MRI, PET scan), postmortem measures	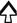 or 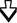	Conditional	(37, 81, 82)

*These are general indicators, and their expression may be species-specific. This table aims to stimulate discussion and consideration of the complexity of positive HAR assessment and our current state of knowledge on its assessment*.

1 *“Conditional”: the presence of this sign could indicate a positive HAR, but may need to be interpreted in conjunction with other indicators or the context (e.g., conflicting motivations)*.

2 *The presence of this sign could indicate a positive HAR, but its absence does not necessarily rule out a positive HAR*.

#### Behavioral Changes

We describe here in a typical chronological order the behavioral changes associated with a positive HAR and their specificity to a positive HAR.

The animal can show signs of anticipation before the interaction takes place in cases when the human interaction is predictable or environmental cues signal the arrival of a human. These could be considered “appetitive” signs, such as pacing, vocalizations, or increased behavioral transitions (83). For example, captive Bottlenose dolphins anticipated interactions with humans, through increased surface looking and spy hopping, and these anticipatory behaviors correlated with their subsequent level of engagement in the interaction (84). These anticipatory signals can nevertheless be ambiguous indicators, as either indicative of positive (excitement) or negative (frustration, for instance, if the delay is too long) states depending on the situation (83).

The first reaction of an animal to the arrival of a human in its environment is an orientation response. The animal typically turns its attention toward the human, possibly using various senses other than vision. The orientation response indicates that the animal notices the presence of the human but is not in itself an indicator of the quality of the HAR because of its potential ambiguous underlying motivations reflecting either a positive (e.g., interest) or negative (e.g., vigilance) state. However, head, ear, and body posture or movement and accompanying behaviors may help to distinguish at least between a negative HAR and a neutral to positive HAR; for example, in cattle, head stays in normal position and ears not erected or even hanging loosely while looking toward the person and ongoing rumination.

Once the human enters the animal's environment, the latency to approach, in the form of voluntary seeking behavior of the animal, is generally an indicator of a positive HAR and/or curiosity. Approach is context-specific (e.g., novelty of the situation and stimulus) and species-specific and therefore should be used with other indicators. However, a lack of approach does not preclude a positive HAR but may just indicate low motivation for (physical) interaction at this time (68); this especially accounts for situations in the home environment where many distractions or competing motivations may occur (e.g., feeding, resting). In many cases, spatial proximity is also a sensitive indicator of a positive HAR, for instance, reflected by the duration of time spent near a human (30, 51).

The number or duration of interactions initiated by the animal is often used as an indicator of the quality of the HAR. Although a quantifiable metric, it does not necessarily reflect the relationship because the animal may modulate the interaction with the human according to its needs; for instance, the animal may want to interact more if it is distressed, may not have interacted for some time, or conversely may not be interested at that time in interacting (68). In this regard, further research is required on refined indicators of interactions (85), such as by studying the complementarity, reciprocity, and synchrony of behavioral exchanges that have been shown to be important in the quality of parent–infant interactions (70). For instance, behavioral synchrony has been shown to be linked to affiliation in humans (86), and locomotor synchrony has been observed between dogs and their owners (69). Further, dogs with lower initial oxytocin levels received more stroking from their owner (87), demonstrating the dynamic interplay of the HAR.

The type of behaviors and body posture displayed during approach and contact with humans, reflecting the animal's level of engagement in the interaction, can provide information regarding the perception and motivation of the animal. In particular, solicitation behaviors such as species-specific grooming solicitation postures and other types of physical solicitation for contact such as touching, nudging, scratching the human with the paw, or vocalizations are indicators of the animal's motivation to engage and can be interpreted as clear signs of a positive perception of the human. Animals may also expose body areas where they wish to be stroked, for example, the ventral neck area in cattle (35, 52), the abdominal area in pigs (37), or the back rather than the head region in dogs (65). These types of behavioral responses, exposing often vulnerable body region, may be interpreted as involving a level of trust reflecting a positive HAR, although some behaviors such as lying with the belly exposed may also indicate submission in dogs, for instance, and therefore do not necessarily indicate a positive HAR. In most cases, these behaviors are similar to those shown during intraspecific sociopositive interactions, although there are some interspecies specific behaviors [e.g., dog vs. wolf (88)].

The response of the animal in the presence of the human is obviously a key indicator of a positive HAR. A lack of avoidance response to humans is usually indicative of low fear of humans (22). Ear position changes or positions (forward vs. side or backward, or erected vs. hanging) have been used to interpret the valence of human contact [sheep (53, 54), cow (52, 55, 56), dog (57), horse (58)], and recent work investigated more subtle changes in facial expression [cat (60), parrot (61)]. Tail wagging in dogs is often cited as an indicator of enjoyment, but it may be a sign of arousal rather than specifically positive valence (59). In some species, some vocalizations are often associated with positive interactions, for instance, purring in cats (46). A rapid reduction in distress vocalizations and increased proximity seeking toward humans can also be interpreted as a positive perception of human presence [goat (75, 76), hand-reared sheep (47, 51)]. Redirected or displacement behaviors [e.g., in dogs yawning, lip- or muzzle-licking, and looking away or toward the ceiling (89)] may be negative indicators, reflecting a reluctance to interact or conflicting motivations. Similarly, pigs that were used to stroking or scratching expressed more high-pitched vocalizations when the handler did not provide gentle tactile contact, which the authors interpreted as indicators of stress possibly resulting from frustration due to the fact that the previously-handled piglets expected positive human contact (90).

Finally, qualitative behavior assessment in which human observers rank the bodily expression of the animals using word descriptors (91) seems promising as a holistic approach for differentiating HAR (62).

Behavioral changes to assess a positive HAR may be species-, individual-, and context-specific (see section Implications for Practice). In particular, the way the human and the animal initiate the contact or interact appear as important modulators of these changes [pig (92), dog (65, 66, 78, 87)].

#### Physiological Changes

In addition to behavioral changes, studies have also shown a wide array of physiological changes linked to human–animal interactions (93).

Oxytocin, in particular, has attracted a lot of attention for its link to social processes. Positive interactions, in particular with familiar humans, generally raise oxytocin concentration [reviewed in (77)]. The relationship between positive HAR and changes in oxytocin concentrations is nevertheless complex and not fully understood [dog (66, 67, 79, 87); sheep (94); domestic species (77); dairy cattle, pig, goat (95)].

Cortisol concentration also changes following positive interactions, with the direction of change reflecting either excitement [dog (79)] or conversely relaxation [dog (66)], and change in cortisol concentration is time- and context-dependent.

Heart rate and heart rate variability measures provide dynamic information on activation of the autonomic response. In general, studies show a reduced heart rate and an increase in measures of parasympathetic activation (e.g., high frequency, or greater root mean square of successive differences) during or after interacting positively with a human [sheep (54); dog (78, 79)], partly dependent on the body region of grooming [horse (96); cow (52)] or the type of interactions (97).

Finally, the involvement of other physiological changes, especially neurotransmitters such as opioids and dopamine and immune parameters, such as immunoglobulin-A require further research. In fact, positive interactions induce an array of physiological and immune changes in both humans and animals (93), and -omics approaches [e.g., transcriptomics, proteomics, metabolomics (98)] could be useful to decipher the biological pathways modulated by positive HAR and its effects on health. For instance, rabbits that received regular positive human contact showed lower incidence of atherosclerosis (99). Gently handled chickens had a higher immune response and disease resistance (100), and mere regular visual contact with humans increased the antibody response to Newcastle disease vaccine and reduced heterophil-to-lymphocyte ratio following capture and restraint later in life (101).

#### Cognitive and Neurobiological Effects

Few studies have focused on the cognitive and neurobiological changes induced by a positive HAR. Cognitive bias tasks have recently been popular as an indirect assessment of emotional states by studying affect-related cognitive changes (102). In rats, tickling by a human induces a more positive judgment of ambiguous cues, suggesting that it induces a positive emotional state (80). Similarly, piglets that experienced positive human contact judged ambiguous cues more positively (40). Conversely, dogs show a more negative judgment of ambiguous cues after being left alone (103). Whether a positive HAR leads to positive emotional states requires further research.

Other approaches have relied on the animal's memory of humans. Pigs can remember positive interactions with humans for at least 5 weeks (32). Horses that were trained using positive reinforcement training with positive human interactions remembered the human 6 months later and spent more time close to the familiar human (63). Sheep can be trained to discriminate sheep and human faces (104) and remember those faces for over 2 years (28), and sheep also recognize their familiar caretaker without any pretraining (64). These findings support that a positive HAR can be long-lasting.

Finally, neurobiological studies of positive HAR are still in the early stage with the use of, for instance, functional near-infrared spectroscopy [dog (81)], electroencephalography [pig (37)], or postmortem brain measures [sheep (82)]. Our understanding of the neuroscience of human–animal interactions could progress with new techniques such as neuroimaging [dog (105)], allowing non-invasive longitudinal neurobiological studies.

#### Postinteraction Changes

Most studies have focused on studying those biological changes when or around the time a human is present. There are also a number of changes that can occur following positive human–animal interactions, that is, at other times than when the animal and human interact. These can be indicative of positive (relaxation or “postconsummatory”) or negative (e.g., separation distress, searching behavior) effects. These effects that outlast the interaction *per se* are often overlooked as compared to the changes occurring during the interaction. Indices of relaxation include hanging ear posture [cattle (55)], lower heart rate [dog (71)], greater parasympathetic activity [various species (72)], elevated brain oxytocin concentration [pig (73)], and shorter latency to rest or better sleep quality [dog (74)].

There can also be indicators of attempts to restore contact, for example, after interruption of an interaction, as evidenced by signs of separation distress or searching behavior [dog (45); hand-reared sheep (47)]. Although these may be signs of distress and negative emotional states, searching behavior and separation distress when an interaction is disrupted are nevertheless signs of a positive HAR.

Further research is warranted on whether a positive HAR can induce baseline biological changes on an animal, for example, changes to its time-budget outside of the interactive sessions with humans. For instance, gentle human interactions during milking or rearing can lead to fewer aggressive interactions between dairy cows once they return to the herd (106) and lower adrenocortical activity in calves (107). Similarly, flocks of gently handled chickens showed fewer agonistic interactions (108).

### Epistemiological Considerations for the Investigation of a Positive Human–Animal Relationship

Motivation and preference tests can be used to assess the HAR (22). They can provide insight into the animal's perception (109), by testing animals on what they find positively and negatively reinforcing; what they want or do not want and how much they value the stimulus. Nevertheless, preferences and motivation may vary with the time of day, environmental conditions, the animal's previous experience, and the current condition and familiarity with the options under study (110), requiring careful interpretation.

The most common tests used for HAR assessment have been the stationary/passive human test, approaching/active human test, and tests involving separation from a human [reviewed in (22)]. We cover below various aspects of the development and use of tests to specifically assess a positive HAR.

An animal voluntarily approaching and interacting (non-aggressively) with a human is a prime indicator of a positive HAR. This is nonetheless not sufficient to qualify as a specific positive HAR indicator because the animal may approach and interact because of curiosity or a motivation to explore. The motivation to explore may also be initially affected by how fear-provoking the situation is. Conversely, the lack of approach is not sufficiently conclusive to reject a positive HAR due to potentially conflicting motivations and a momentary lack of motivation for interaction.

Tests based on avoidance responses (e.g., distance of withdrawal by the animal from an approaching human) are often used to measure the fear dimension of the HAR. However, acceptance of approach and subsequent touch and stroking by a human are clear indicators of a positive HAR and can be more sensitive in differentiating the quality of HAR than approach behavior toward a stationary person [pig (36, 92), cattle (35, 68, 111)]. The sensitivity of the tests nevertheless depends on the species tested and contextual features (22), as well as phenomena such as generalization of the response toward unfamiliar humans.

Situations where there is a lack of control offered to the animal because the animal is restrained or limited to a constrained space or when contact is imposed on the animal without the possibility for the animal to avoid or withdraw may influence the validity of the HAR assessment. Nevertheless, the few studies to date comparing restrained and unrestrained animals showed relatively similar responses to humans (68, 112, 113). Standardized interactions by the humans, such as imposing contact on the animal or using highly standardized interactive features (e.g., predetermined interaction in terms of bout frequency or duration) are commonly used in research settings as they provide experimental control. However, free-choice interactions may replicate real-life situations more faithfully because control over the situation may be linked to the perception of the situation, although this hypothesis remains to be tested. It may be important that the animal is provided with a sense of control or agency (114) by free-choice approach about when and how to interact (61, 78). This is similar to the case for second-person neuroscience (85) that emphasizes the need to look at situations of active social engagement and reciprocal behaviors, rather than passive observation or being subjected to a situation with a lack of agency. This argument is based on the fact that an interaction typically involves active participation from both agents.

The test should be conducted in an appropriate environment. Animals have been most often tested individually, which may not reflect their typical reaction when in their social group. Furthermore, testing environments have most often been barren, offering few choices other than interacting with the human. Hence, this questions the specificity and validity of the animal's response toward the human as an indicator of a positive HAR in cases where there is a lack of choice (110).

The experience of the animal with humans is obviously crucial to consider, as additional positive interaction treatments may fail to show additional effects if the HAR is already positive (107, 115). Hence, it is important to assess the “baseline” HAR in the animal's real-life environment (i.e., outside of the experimental treatments) and take into account the ratio of negative and positive human contact (106).

Many studies, to date, compared positive and negative human interaction treatments, but lacked a control treatment [e.g., (73, 74, 116)]. This control treatment usually consists of minimal human contact involved in routine care and management (117), or human present with no active interactions (52). It is crucial to demonstrate that the HAR is specifically positive, rather than neutral. If comparing only positive and negative interactions without a control treatment, a potential difference may be induced by negative treatment effects without being able to distinguish them from the positive treatment effects.

As mentioned earlier, more detailed analysis of the interaction could assist in assessing the quality of the HAR, for instance, based on the synchrony between partners (69), or the functional complementarity of the exchange and/or responsiveness using similar approaches to those used in humans (70).

## Implications for Practice

### Developing and Cultivating a Positive Human–Animal Relationship: How?

The HAR is a dynamic and reciprocal process modulated by individual and contextual features. An understanding of its development and regulatory mechanisms provides practical opportunities to develop and maintain a positive HAR for animal caretakers.

Gentle handling is particularly effective [sheep (6), pig (32, 92), ostrich (118)], although passive human presence may be required initially to habituate the animal [pig (36)]. Note that some species may not need physical contact, and visual contact may be sufficient [e.g., poultry (119, 120)], although the need for and type of contact are strongly species-dependent. Positive interactions involve several species-specific sensory channels: tactile, visual, auditory, and olfactory, and are often multimodal [dairy cow (121), sheep (82), pig (23)]. In many species, brief (from 15 s to a few minutes) opportunities to interact with humans over days or several weeks are sufficient to reduce the animal's fear of humans and encourage approach and interaction [dog (33), horse (122), cattle (123), pig (92, 124), poultry (120)], suggesting that a positive HAR from the perspective of the animal can develop rapidly. Studies examining tickling of rats on positive affective states have demonstrated the importance of the dosage and characteristics of this technique (125). Further research is needed to determine the minimal “dose” of human contact required to form a positive HAR in terms of type, frequency, and duration of interaction.

Incorporating training principles, primarily through the use of positive reinforcement, has been broadly and successfully used in practice for zoo and companion animals to improve handling by reducing the aversiveness of some procedures [dog (126), cat (127), horse (63), primates (128)]. Training is not yet commonly used in farm settings despite proof of its effectiveness in research settings [pig (129, 130), sheep (131), cattle]. Given that human contact *per se* can be perceived as inherently rewarding, it could be used as a reward during training (e.g., stroking, brushing, playing), although food rewards may facilitate this process.

It may be easier to develop a HAR with young animals [dog (132), pig (124)] because they may had fewer negative experiences with humans, have greater learning ability (133) and higher levels of curiosity and exploration (26, 50) than adult animals. In particular, the development of a HAR may be most effective during sensitive periods for socialization such as during early life (49) or socially stressful periods such as after weaning (134). Social facilitation, building on the transmission of the HAR with the dam or other conspecifics, can also be effective [horse (135), sheep (26), pig (136)]. There is even evidence of transgenerational transmission of positive HAR, as human contact altered mother quails' egg physiological environment and led to less emotionally reactive offspring (137).

Familiarity and previous experience with humans can influence the HAR. Nevertheless, if the animal's experience with familiar humans is mainly positive, domestic animals can generalize their positive response toward unfamiliar humans [sheep (138–140), dog (141, 142), pig (8, 10, 36), horse (143), cattle (34)], although the animal may still prefer familiar over unfamiliar humans [sheep (116)]. Generalization of the HAR to unfamiliar humans depends not only on past experiences but also familiarity of the context such as the behavior or other characteristics of the human and the location [cattle (42, 144, 145), pig (32, 36, 90, 146)], which may affect the motivation to approach and remain near an unfamiliar human. As such, a positive HAR is not necessarily limited to a personalized one, that is, toward a specific human.

It is important to keep in mind the potential modulating effects on the HAR due to genetics and species differences [fox (147), dog (9), sheep (41)], individual differences [dog (78)], previous experience and age [pig (32, 36)], social context [cattle (148), sheep (26), pig (136)], and other context-specific aspects.

In addition, the attitudes, skills, and knowledge of humans influence their behavior toward animals and in turn the animal's perception of humans (1, 5). Although beyond the scope of this article, these human factors should be considered when thinking of the HAR. There is also increasing evidence that animals can recognize human facial expression of emotions [dog (149–151), horse (152), goat (153)] or human bodily expression [cat (154)] and prefer positive human emotional expressions.

The predictability of the interaction can strongly affect the animal's response to humans [pig (30), beef cattle (144), sheep (116), dog (155)], because as mentioned previously the relationship is based on the animal's expectation of its interaction with humans. The HAR concept implies the predictability of human–animal interactions. In addition, the provision of choice and control available to the animal in terms of when and how to interact appears to be important [dog (79), pig (92), cattle (11)].

A key aspect for the human is to pay attention to the animal's response to humans. A positive HAR can be assessed based on behavioral observations as highlighted previously (see section Assessment of a Positive Human–Animal Relationship), and as such it is feasible to cultivate a positive HAR in practice based on this knowledge and without the need for specific equipment.

### The Benefits of a Positive HAR: Why?

**Figure 1:**
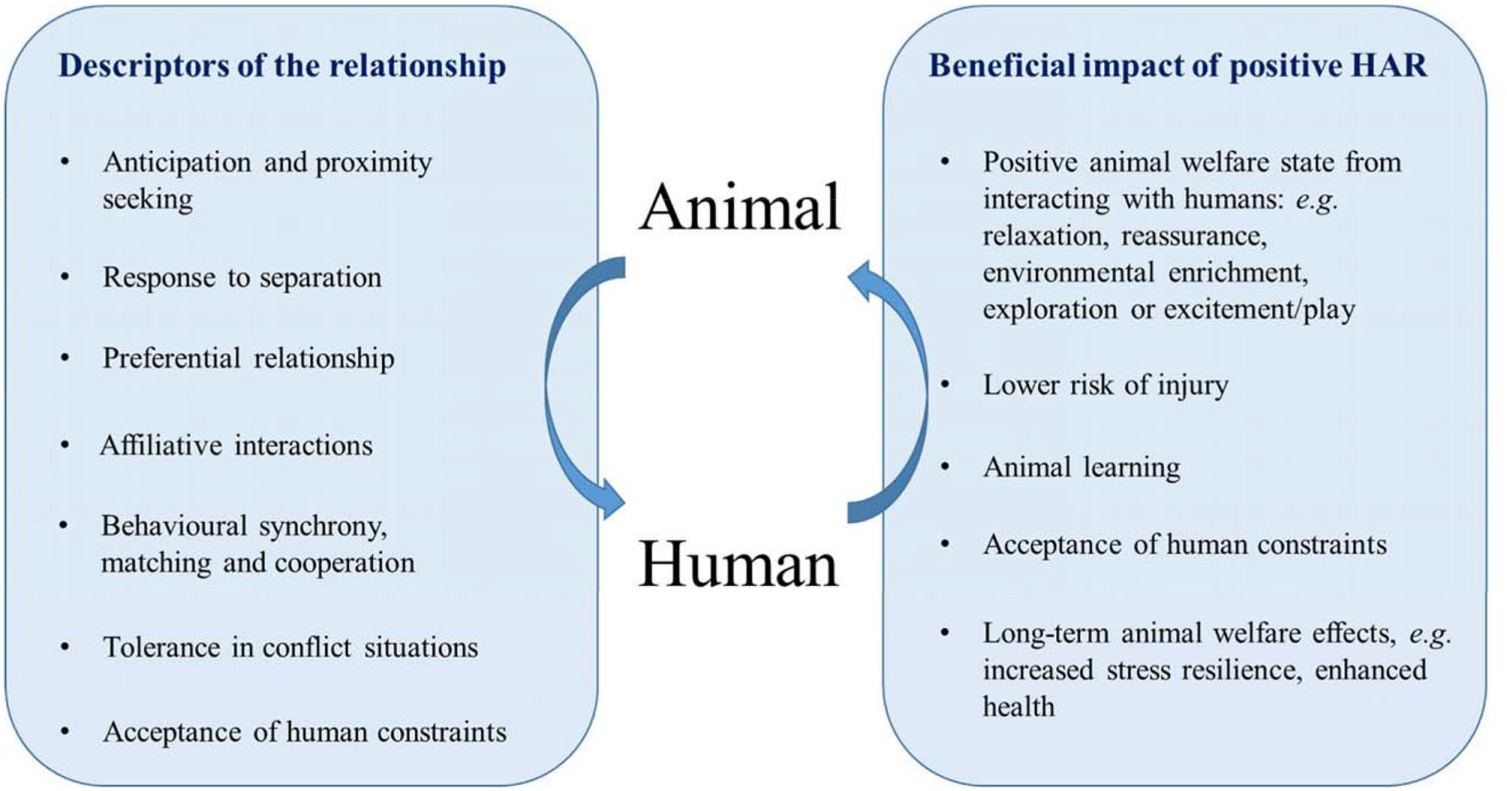
The different dimensions of a positive human–animal relationship for the animal. The arrows symbolize the interactions between the animal and the human.

The HAR can have important and long-lasting effects on the welfare of animals, and this relationship is often critical to the domestic animals' role, for example, animal productivity and ease of handling and management, as well as companionship and satisfaction for the human. Evidence is accumulating on the potential welfare benefits of a positive HAR (Figure 1).

There are benefits of a positive HAR on stress resilience. For example, offering positive interactions to shelter dogs can reduce their cortisol level (156) and combined with training increases adoptability (157). Walking and stroking shelter dogs for 15 min once a week for 6 weeks increased the time they spent visible from the front of the pen and tail wagging (158). Only three 10-min bouts of handling was sufficient for shelter dogs to show a preference for the handler (33). Five minutes of weekly brushing dairy heifers facilitated their acclimation to the milking routine (123). Five seconds of back scratching of sows for 1 week prior to farrowing reduced piglet mortality in sows (159), although this stroking treatment was confounded with music from a radio. These examples indicate that brief positive interactions with humans can benefit animal management and animal welfare.

A positive HAR can also buffer aversive procedures where humans are involved such as veterinary inspections or management interventions [sheep (131), pig (129), cow (160), ostrich (118)], presumably by removing human-related stress-eliciting components. In addition, humans can provide social support to animals during stressful times, especially for animals kept in suboptimal social environment [pig (161), sheep (47), chimpanzee (17)]. Stroking by the owner calms the behavioral and heart rate responses of dogs to subsequent separation (71). The effectiveness of providing social support can be modulated by the quality of the HAR [dog (66, 162)].

As an enrichment strategy, positive interactions with humans present several advantages as they usually occur daily and can be combined with routine checks, can be manipulated for their predictability to minimize habituation, and do not require additional resources (e.g., material). For example, orangutans preferred to stay in the part of their zoo enclosure where they can be close to and observe visitors (163), suggesting that interactions with humans may be enriching for them.

There is limited direct evidence to date that a positive HAR stimulates positive affective states in domestic animals (19). Tickling of rats (80) or gentle contact of pigs (40) by humans induces more positive judgment of ambiguous cues, suggestive of a positive emotional state. Positive or negative human interactions influence the sleeping patterns of dogs (74), although in the absence of a control treatment it remains to be determined whether this was the result of the positive or negative interactions.

Developing a positive HAR provides benefits in the long term. The persistence of the effect of early positive human contacts [5–16 weeks, pig (32, 124); 6 months, dairy cattle (11); 6–8 months, beef cattle (134); 24 months (164); 8 months, goat (76); 25 months, (165); 6–8 months, horse (63)] makes it an intervention with potentially long-lasting effects. Nevertheless, there may also be risks or disadvantages of a positive HAR. For example, pigs that experienced positive human interactions can be difficult to handle in familiar locations because of low fear of humans (166); however, pigs that are fearful of both humans and the unfamiliar handling location take longer to move and balk more than pigs that have experienced positive human interactions (146), suggesting an interplay between the HAR and the familiarity of the environment. It is also important to keep in mind trust and safety of both partners, because animals with low fear of humans can be dangerous, especially in case of inappropriate human behavior as it is often the case in dog bites of children (155) or during risky or potentially aversive procedures that involve close contact or handling [horse (25), dairy cow (121, 167)]. In order to be able to both manage the animals in a practicable manner and minimize the risks of aggression or injuries, a positive HAR may benefit from settings boundaries such as respecting a safe distance and avoiding potentially dangerous interactions. Social animals usually learn to distinguish acceptable from unacceptable social behaviors during their development as part of the socialization process and the development of their social skills, and this socialization process may also affect the animal's behavior toward humans.

Hence, a positive HAR can provide animals with positive welfare outcomes (20, 168), such as greater stress resilience, social support, environmental enrichment, possibly positive affective states, as well as benefits to their role for humans.

## Conclusions

Positive experiences with humans lead to domestic animals seeking and interacting with humans. Consequently, a positive HAR can bring intrinsic rewards to the animal. It can be used to elicit positive emotions and other positive welfare outcomes. Nevertheless, our understanding of the underlying processes that govern the positive perception of humans by animals is incomplete and will benefit from further research, especially in regard to the type, frequency, and length of human interaction necessary to establish an effective positive HAR. In particular, the importance of providing animals with a sense of agency and its effect on the HAR remains poorly understood. Further research is needed to identify how much changes in features of interaction reflect the quality of the relationship.

## Author Contributions

J-LR wrote the first draft of the manuscript and drafted Table 1. XB drafted Figure 1. SW, XB, and PH edited the manuscript. All authors reviewed and approved the0 final manuscript.

## Conflict of Interest

The authors declare that the research was conducted in the absence of any commercial or financial relationships that could be construed as a potential conflict of interest.
